# Combined effect of hyperfiltration and renin angiotensin system activation on development of chronic kidney disease in diabetic db/db mice

**DOI:** 10.1186/1471-2369-15-58

**Published:** 2014-04-04

**Authors:** Stella P Hartono, Bruce E Knudsen, Lilach O Lerman, Stephen C Textor, Joseph P Grande

**Affiliations:** 1 Department of Laboratory Medicine & Pathology, Mayo Clinic, 200 First Street SW, Rochester, MN 55905, USA; 2 Medical Scientist Training Program, Mayo Clinic, 200 First Street SW, Rochester, MN 55905, USA; 3 Division of Nephrology & Hypertension, Mayo Clinic, 200 First Street SW, Rochester, MN 55905, USA

**Keywords:** Diabetes, Hypertension, Renal artery stenosis, Renovascular hypertension, Inflammation

## Abstract

**Background:**

Hypertension is a major risk factor for renal disease progression. However, the mechanisms by which hypertension aggravates the effects of diabetes on the kidney are incompletely understood. We tested the hypothesis that renovascular hypertension accelerates angiotensin-II-dependent kidney damage and inflammation in the db/db mouse, a model of type II diabetes.

**Methods:**

Renovascular hypertension was established in db/db and wild-type control mice through unilateral renal artery stenosis (RAS); the non-stenotic contralateral kidneys evaluated 2, 4 and 6 weeks later. Angiotensin-II infusion (1000 ng/kg/min), unilateral nephrectomy, or both were also performed in db/db mice to discern the contributions of hypertension versus hyperfiltration to development of chronic renal injury in db/db mice with RAS. The effect of blood pressure reduction in db/db mice with RAS was assessed using angiotensin-receptor-blocker (ARB) or hydralazine treatment.

**Results:**

Db/db mice with renovascular hypertension developed greater and more prolonged elevation of renin activity than all other groups studied. Stenotic kidneys of db/db mice developed progressive interstitial fibrosis, tubular atrophy, and interstitial inflammation. Contralateral kidneys of wild type mice with RAS showed minimal histopathologic abnormalities, whereas db/db mice with RAS developed severe diffuse mesangial sclerosis, interstitial fibrosis, tubular atrophy, and interstitial inflammation. Db/db mice with Angiotensin II-induced hypertension developed interstitial lesions and albuminuria but not mesangial matrix expansion, while nephrectomized db/db mice exhibited modest mesangial expansion and interstitial fibrosis, but not significant albuminuria. The combination of unilateral nephrectomy and angiotensin II infusion reproduced all the features of the injury albeit in a less severe manner. ARB and hydralazine were equally effective in attenuating the development of mesangial expansion in the contralateral kidneys of db/db mice with RAS. However, only ARB prevented elevation of urinary albumin/creatinine in db/db mice with RAS.

**Conclusion:**

Renovascular hypertension superimposed on diabetes exacerbates development of chronic renal disease in db/db mice at least in part through interaction with the renin-angiotensin system. Both ARB and hydralazine were equally effective in reducing systolic blood pressure and in preventing renal injury in the contralateral kidney of db/db mice with renal artery stenosis. ARB but not hydralazine prevented elevation of urinary albumin/creatinine in the db/db RAS model.

## Background

Diabetic nephropathy is the leading cause of end-stage renal disease in the United States. In 2008, 44% of new cases of kidney failure were attributed to diabetes, and the numbers are expected to increase as the number of Americans with diagnosed diabetes has reached above 20 million – with another estimated 7 million people with undiagnosed diabetes [1].

Hypertension is a major risk factor for renal disease progression in patients with diabetes. One of the most common causes of secondary hypertension is renal artery stenosis (RAS). Atherosclerosis, the main cause of RAS, shares many similar risk factors with diabetes type II, thereby making it likely for RAS to co-exist in diabetic type II patients. Indeed, in patients with type II diabetes and hypertension the incidence of RAS is between 17-44% [2-4] and even subcritical RAS confers a significant risk for progression to renal failure [3]. However, it is still unclear if this increased risk is due to hypertension alone or contributed by other factors that are induced during RAS. It is well recognized that RAS is associated with activation of the renin-angiotensin system which leads to systemic hypertension. We have previously demonstrated that in our unilateral RAS model, the decrease in blood flow to the stenotic kidney is associated with an increase in blood flow to the contralateral kidney [5], raising the possibility that the contralateral kidney may be susceptible to hyperfiltration injury. However, few studies have directly addressed potential interactions between hyperfiltration and pathophysiologic activation of renin-angiotensin system in the development of diabetic renal disease.

We therefore sought to test the hypothesis that activation of the renin-angiotensin system and hyperfiltration interact to produce chronic injury in the contralateral, non-stenotic kidney of db/db mice. We demonstrate that db/db mice with RAS develop diffuse mesangial sclerosis in their contralateral kidney that is not observed in age matched db/db mice or in WT mice with RAS. Unilateral nephrectomy, infusion of Angiotensin II, or their combination in age-matched db/db mice failed to reproduce the glomerular and, in particular, the interstitial lesions observed in db/db mice subjected to RAS. Prophylactic administration of hydralazine and valsartan yield only modest attenuation of renal damage in the contralateral kidney of db/db mice with RAS, with no difference between the two interventions. We conclude that renovascular hypertension in diabetic db/db mice produced accelerated and progressive renal injury that cannot be explained by increase in blood pressure alone.

## Methods

### Animal models

C57BLKS (WT) (N = 50) and C57BLKS/JLepr (db/db) (N = 166) male mice, 5–6 weeks old, were obtained from Jackson Laboratory (Bar Harbor, ME). Induction of hypertension and RAS was performed using a modified cuff approach as previously described [5,6] at 6–7 weeks of age (N = 10 for each time points). Mice were studied at 2, 4 and 6 weeks post RAS induction. Sham surgeries (N = 5 for each time points) consisted of a flank incision and mobilization of the renal artery without placement of a cuff. To determine the effect of angiotensin II induced hypertension with or without hyperfiltration, unilateral nephrectomies or sham surgeries [7,8] were performed on db/db mice at 6–7 weeks of age as previously described. Osmotic mini-pump (Alzet model 2004, Durect, Cupertino, CA) loaded with Angiotensin II (1000 ng/kg/min) or PBS were inserted subcutaneously on the same day [9-13]. To determine the effect of lowering blood pressure, Hydralazine (10 mg/kg/day) or angiotensin II receptor blocker Valsartan (8 mg/kg/day) was administered in drinking water of db/db mice with RAS on the day of the surgery (N = 10 per each treatment, N = 8 for vehicle treatment).

Blood pressures were measured on conscious acclimatized mice using tail cuff method (CODA System, Kent Scientific, Torrington, CT) three days prior to surgery and subsequently at two-week intervals. Mice were euthanized by exsanguination at 2, 4, and 6 weeks post-surgery. Kidneys and hearts were perfused with sterile PBS, excised, weighed, and either preserved immediately for histology, or shock frozen in liquid nitrogen for Western blotting and PCR analysis. All animal protocols were approved by the Mayo Clinic Institutional Animal Care and Use Committee.

### Biochemical analysis

Blood was collected by tail bleed for serial measurements and finally by terminal bleed. The plasma fraction was separated by centrifugation upon collection and stored at -80°C until assay. Renin activity in plasma was assessed via production of angiotensin I from angiotensinogen using a commercially available GammaCoat Plasma Renin Activity 125I RIA kit (DiaSorin, Stillwater, MN), using porcine angiotensinogen (A2283, Sigma-Aldrich, St. Louis, MO) substrate. Urine albumin and creatinine were measured on spot urine sample using Albuwell and Creatinine kit (Exocell, Philadelphia, PA). Commercially available ELISA kits were used for the measurements of serum CCL2 and IL-6 (R&D Systems, Minneapolis, MN).

### Histology and immunohistochemistry

Kidneys were fixed with 10% neutral buffered formalin and processed for histology or immunostaining using standard techniques. Histological section (5 μm thick) were prepared and stained with hematoxylin-eosin (H&E), Masson’s trichrome, periodic acid-Schiff, anti-fibronectin (Abcam, Cambridge, MA), and anti-F4/80 (AbD Serotec, Raleigh, NC). H&E slides were used to assess atrophy, glomeruli area and diameter. Atrophy was semi quantitatively assessed by a renal pathologist by assessing the relative surface area occupied by atrophic tubules compared to the total cortical surface area, as previously described [14]. Mesangial matrix expansion was assessed in PAS sections with a 0–4 scale (0 = normal, 1 = mild mesangial matrix expansion, 2 = moderate matrix expansion with patent capillaries, 3 = severe matrix expansion with segmental capillary loop consolidation, 4 = severe matrix expansion with global capillary loop consolidation). Each glomerulus was scored positive or negative for fibronectin, and quantified as percent positive glomeruli over total glomeruli per tissue sections. Degree of fibrosis was quantified in trichrome sections by assessment of ratio of surface area of the cortical area (avoiding great vessels and glomeruli) at 200× magnification. Interstitial fibronectin deposition and renal microphage infiltration was similarly quantified in fibronectin and F4/80 sections respectively. All measurements and quantification were performed in a random blinded fashion using an Olympus BX50 microscope (Olympus America, Melville, NY), a Micropublisher 3.3 RTV camera (Q-Imaging, Surrey, BC), and the NIS Elements Imaging Software (Nikon Instruments, Inc., Melville, NY).

### Transmission electron microscopy

For transmission electron microscopy, tissue was removed from the paraffin block and placed into warm xylene for 90 minutes, transferred to warm absolute ethanol for 30 minutes, then transferred to decreasing concentrations of ethanol to 60% then placed into Trump’s fixative (1% glutaraldehyde and 4% formaldehyde in 0.1 M phosphate buffer, pH 7.2) for overnight fixation. Tissue was then rinsed in 0.1 M phosphate buffer, pH 7.2, post-fixed in 1% osmium tetroxide for one hour, rinsed in distilled water, dehydrated, embedded in Spurs resin, and sectioned at 90 nm. Micrographs were taken on a Philips Technai 12 (FEI, Hilsboro, OR) operating at 80KV. Glomerular basement membrane measurement was performed by Mayo Clinic Electron Microscopy Core Facility in a random blinded fashion.

### mRNA analysis

Total RNA was extracted with RNeasy Mini Plus kit (Qiagen, Valencia, CA) and reversed transcribed using iScript cDNA synthesis kit (Bio-Rad, Hercules, CA). Gene expression analysis was determined by quantitative real-time PCR using CFX96 (Bio-Rad, Hercules, CA) and normalized to *18 s*. The following primers were used: *Ren1* forward 5’ –GAG GTA GCG ACC CGC AGC ATT AT- 3’; *Ren1* reverse 5’ – GCG CTG CCT CCC AGG TCA AA- 3’; *Ccl2* forward 5’ – AGC ACC AGC ACC AGC CAA CTC – 3’; *Ccl2* reverse 5’ – TGG ATG CTC CAG CCG GCA ACT – 3’; *Il-6* forward 5’ – TGG TGA CAA CCA CGG CCT TCC – 3’; *1 l-6* reverse 5’ – TAA GCC TCC GAC TTG TGA AGT GGT – 3’; *18 s* forward 5’ – CTC AAC ACG GGA AAC CTC AC – 3’; *18 s* reverse 5’ – CGC TCC ACC AAC TAA GAA CG – 3’.

### Statistical analysis

Data are presented as mean ± SE. Comparisons between two groups were done using student t-test for parametric data and Mann–Whitney test for non-parametric data or data without normal distribution. To assess interactions between time points and multiple groups, two-way ANOVA followed by a Tukey adjustment for post-hoc comparison across different time points and treatment groups was used. For comparison across multiple groups, one-way ANOVA followed by a Tukey adjustment was used for post-hoc comparison of the measurements. P values <0.05 were considered significant. Statistical analyses were performed with Graphpad Prism 6 (GraphPad Software, La Jolla, CA).

## Results

### Wild type (WT) and db/db mice with RAS develop similar degree of hypertension

**Figure 1:**
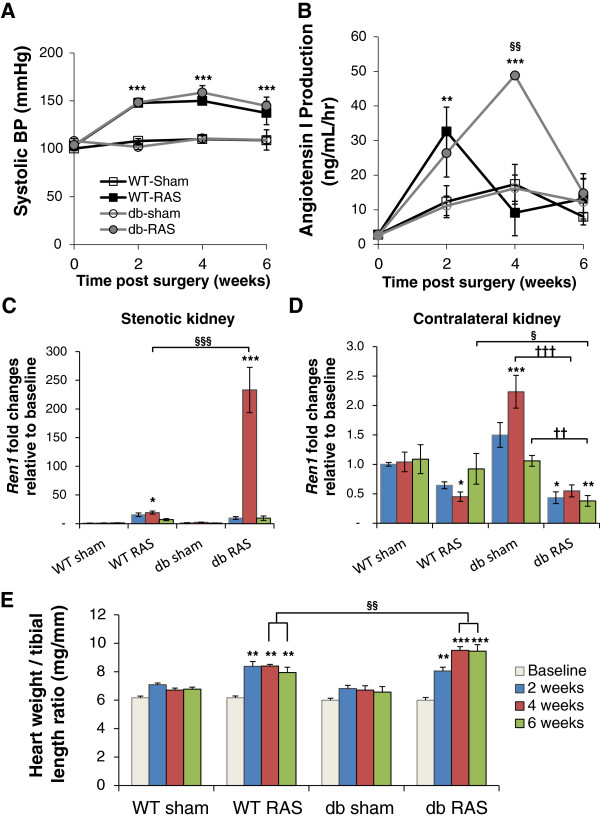
**Db RAS and WT RAS mice developed similar degree of hypertension. A**. Systolic blood pressure (BP) of db and WT mice subjected to RAS or sham surgery. Both WT RAS and db RAS experienced similar elevation of systolic BP (WT RAS vs. db RAS, p = ns) compared to their sham control. **B**. Plasma renin activity in the db and WT mice subjected to RAS or sham surgery as measured by Angiotensin I production. **C-D**. RT-PCR analysis of Ren1 mRNA as normalized to 18 s in stenotic **(C)** and contralateral kidneys **(D)** of db and WT mice subjected to RAS or sham surgery, relative to baseline. **E**. Ratio of heart weight to tibial length of db and WT mice subjected to RAS or sham surgery. Data are presented as means ± SE. * p < 0.05, ** p < 0.01, *** p < 0.001 vs. WT sham. §§ p < 0.01, §§§ p < 0.001 vs. WT RAS. †† p < 0.01, ††† p < 0.001 vs. db sham. Statistical significance was determined by 2-way ANOVA followed by Tukey adjusted post-hoc comparison.

To determine the effect of renovascular hypertension on the development of diabetic nephropathy in the diabetic db/db mouse, we subjected db/db and wild type mice to unilateral RAS surgery (db RAS and WT RAS) or to sham surgery (db sham and WT sham). WT and db/db mice had similar baseline systolic blood pressure prior to RAS surgery. Both db RAS and WT RAS experienced a similar increase in systolic blood pressure 2 weeks post-surgery that peaks at 4 weeks and remains elevated at 6 weeks (Figure 1A). WT RAS and db RAS mice had similar increases in plasma renin activity at 2 weeks (Figure 1B). However, while plasma renin in WT RAS mice returned to baseline levels after 4 weeks, plasma renin in db RAS mice was further increased at 4 weeks before going back to baseline levels at 6 weeks (Figure 1B).

To determine whether this increase in renin activity was due to increased renin production or increased enzyme activity, we performed RT-PCR analysis of *Ren1* expression in the stenotic (Figure 1C) and contralateral kidneys (Figure 1D). As expected, induction of Ren1 was much greater in the stenotic kidney (Figure 1C) than the contralateral kidney (Figure 1D). At 2 weeks, *Ren1* expression was increased by 15-fold in the stenotic kidney of WT RAS (p = 0.026 vs. baseline) and increased by 10-fold in the db RAS (p = 0.289 vs. baseline). At 4 weeks, *Ren1* mRNA levels did not further increase in WT RAS mice, but was further induced by 150-fold in db RAS mice (p < 0.001 vs. baseline, p < 0.001 vs. WT sham, WT RAS, or db sham). At 6 weeks, renal *Ren1* mRNA levels approached baseline levels in both WT RAS and db RAS (Figure 1C). As expected, *Ren1* expression in the contralateral kidney of WT RAS and db RAS was similarly down regulated at 4 weeks (Figure 1D). Although *Ren1* expression in the WT RAS mice returned to baseline level by 6 weeks, *Ren1* expression in the contralateral db RAS kidney remained down regulated (p < 0.001 vs. baseline, p < 0.001 vs. WT sham, WT RAS, or db sham).

The hearts of both WT RAS and db RAS underwent hypertrophy, as evidenced by a 15% increase in heart weight to tibial length ratio at 2 weeks following surgery (Figure 1E). However, the hearts were larger in db RAS mice compared to the WT RAS mice at 4 and 6 weeks. Therefore, development of RAS in both WT and db/db mice was associated with renovascular hypertension, increased plasma renin content, increased renal Ren1 expression, and cardiac hypertrophy. After 4 weeks, the increase in plasma renin activity, renal *Ren1* expression, and cardiac hypertrophy were greater in db/db mice than in WT mice subjected to RAS.

### The contralateral kidney of db-RAS mice develops accelerated and progressive renal injury

Although the stenotic kidney of db/db mice developed severe atrophy, the glomeruli appeared to be protected from development of diffuse mesangial sclerosis – an early manifestation of diabetic nephropathy – in accordance with previous reports on the stenotic kidney of diabetic patients (data not shown) [15]. Instead, the stenotic kidney of db/db mice developed tubular atrophy to an extent similar to that observed in the stenotic kidney of WT mice at all time-points (Table 1).

**Table 1:** Percent atrophy of the stenotic kidney of wild type (WT) and diabetic (db) mice with RAS at 2, 4, and 6 weeks post-surgery

**Time post-surgery**	**% atrophy**		**p value**
	**WT RAS**	**db RAS**	
2 weeks	88 ± 6	74 ± 9	0.278
4 weeks	91 ± 2	88 ± 4	0.378
6 weeks	85 ± 7	96 ± 1	0.173

**Figure 2:**
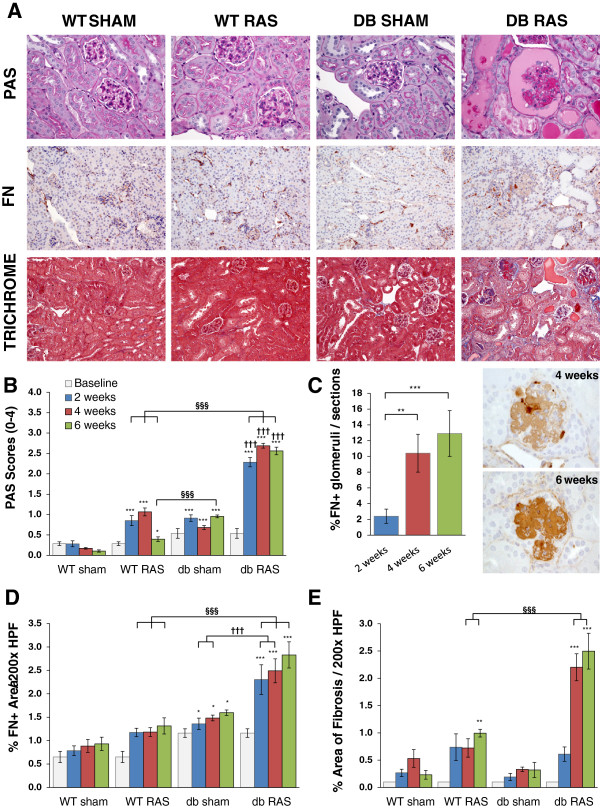
**Contralateral kidney of db RAS but not WT RAS or db sham mice developed chronic renal injury. A**. Representative histological images of contralateral kidney at 4 weeks post-surgery as stained with periodic acid schiff (PAS), fibronectin (FN), and Masson’s Trichrome. PAS images are taken at 400× magnification. Fibronectin and trichrome images are taken at 200× magnification. **B**. Glomeruli of db RAS develop greater mesangial matrix expansion compared to both WT RAS and db sham mice at all time-points. Mesangial matrix expansion was assessed using the PAS sections with a 0 to 4 scale (see Methods). **C**. db RAS but not WT RAS mice develop de-novo fibronectin deposition within the glomeruli. Graph showed % fibronectin positive glomeruli to total number of glomeruli per histological sections in the db RAS mice. No fibronectin positive glomeruli were observed in the WT sham, WT RAS, or db sham. **D**. Interstitial fibronectin deposition is increased in db RAS compared to WT RAS or db sham mice at all time-points. Interstitial fibronectin deposition was assessed as percent area positive for fibronectin in each 200× field over the whole cortical areas. **E**. Interstitial fibrosis is increased to a greater extent in db RAS mice compared to WT RAS mice. Interstitial fibrosis was assessed using trichrome stained sections at 200× magnification. Data are presented as means ± SE. * p < 0.05, ** p < 0.01, *** p < 0.001 vs. WT sham. § p < 0.05, §§ p < 0.01, §§§ p < 0.001 vs. WT RAS. ††† p < 0.001 vs. db sham. Statistical significance for **B**, **D**, and **E** was determined by 2-way ANOVA followed by Tukey adjusted post-hoc comparison. Statistical significance for **C** was determined by one way ANOVA followed by Tukey adjusted post-hoc comparison.

As we previously described, the contralateral kidney in WT mice showed mild glomerular enlargement, with no significant interstitial fibrosis, tubular atrophy, or interstitial inflammation [5,16]. In striking contrast, the contralateral kidney of db RAS mice developed glomerular mesangial matrix expansion that was significantly greater than the contralateral kidney of WT RAS or db sham, as assessed in PAS-stained sections (Figure 2A, 2B) and *de novo* glomerular fibronectin deposition (Figure 2A, 2C). These histopathologic alterations were observed by 2 weeks following RAS surgery (Figure 2B, 2C) mostly at the juxtamedullary glomeruli. At all time-points beyond baseline, the severity of diffuse mesangial sclerosis in the contralateral kidney of db RAS mice was significantly greater than that observed in the contralateral kidneys of db sham mice or in WT RAS mice (Figure 2B).

**Figure 3:**
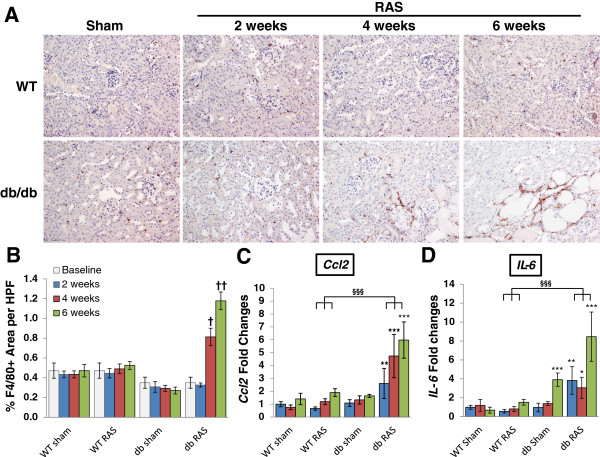
**The contralateral kidneys of db RAS but not WT RAS mice develop progressive inflammation. A**. Representative immunostaining of renal macrophages in paraffin section of contralateral kidneys. Macrophages were stained with anti F4/80 antibody. All images are taken at 200× magnification. **B**. Quantitative analysis of F4/80+ macrophages showed that despite lower baseline F4/80, there was increased infiltration in the contralateral kidney of db RAS mice but not WT RAS mice. **C-D**. RT-PCR analysis of *CCL2***(C)** and *IL-6***(D)** mRNA normalized to *18 s* showed that contralateral kidney of db RAS mice produces more inflammatory cytokines and chemokines compared to WT RAS and db sham. Data are presented as means ± SE. † p < 0.05, †† p < 0.01 vs. db sham. * p < 0.05, ** p < 0.01, *** p < 0.001 vs. WT sham. §§§ p < 0.001 vs. WT RAS. Statistical significance was determined by 2-way ANOVA followed by Tukey adjusted post-hoc comparison to determine corrected p-values.

In addition to the glomerular lesions, the contralateral kidney of db RAS mice developed progressive interstitial fibrosis (Figure 2A, 2D, 2E) significantly greater than that of db sham mice, WT RAS, or WT sham mice at the same time point (Figure 2D). Similar patterns were observed in sections stained for the extracellular matrix proteins fibronectin (Figure 2E). The extent of inflammation in the contralateral kidney as measured by F4/80+ area was also greater in the db RAS mice compared to both WT RAS and db sham mice (Figure 3A, 3B). We then performed RT-PCR to measure the level of chemokine (C-C motif) ligand 2 (*Ccl2*) and interleukin 6 (*Il-6*) mRNA in the contralateral kidney (Figure 3C, 3D). Both were elevated in the contralateral kidney of the db RAS mice in comparison to both WT RAS and db sham mice, indicating presence of inflammation that was not apparent in either the WT RAS or the db sham.

**Figure 4:**
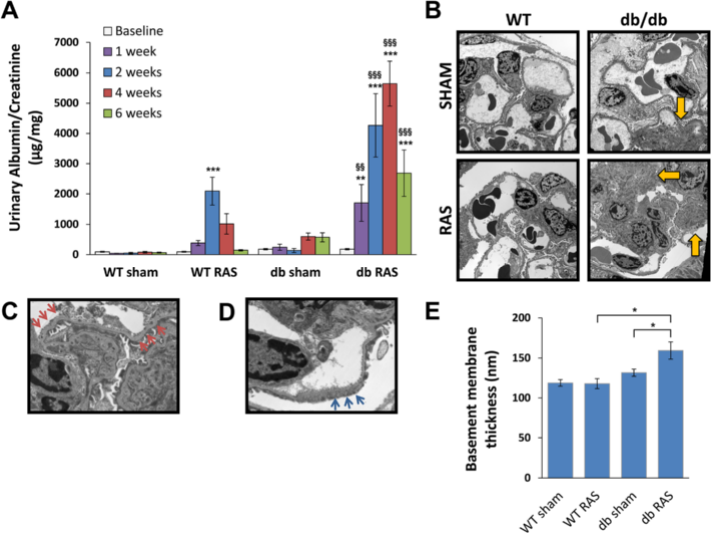
**Db RAS mice developed albuminuria to a greater extent that the WT RAS mice. A**. db RAS experienced persistent and progressive albuminuria compared to the transient albuminuria experienced by WT RAS. Albuminuria was measured in spot urine sample and normalized to urine creatinine. Data are presented as means ± SE. ** p <0.01, *** p < 0.001 vs. WT sham with the same time point. §§ p < 0.01, §§§ p < 0.001 vs. WT RAS or db sham with the same time point. Statistical significance was determined by 2-way ANOVA followed by Tukey adjusted post-hoc comparison. **B-D**. Representative electron microscope (EM) images of contralateral kidneys at 6 weeks post-surgery. db RAS mice showed significant area of mesangial matrix expansion (yellow arrows) **(B)** in comparison to WT RAS or db sham, along with thickened basement membrane (red arrows) **(C)** and podocyte fusion (blue arrows) **(D)**. These changes were not observed in the WT mice (with or without RAS) or db sham mice. Images in **B** are taken at 2900× magnification while **C** and **D** are taken at 9600× magnification. **E**. Morphometric analysis showed significant increase in basement membrane thickness of db RAS compared to WT RAS and db sham. Data are presented as means ± SE. * p < 0.05. Statistical significance was determined by one-way ANOVA followed by Tukey adjusted post-hoc comparison.

WT RAS mice, but not WT sham mice, developed transient albuminuria that persisted up to 4 weeks post-surgery before returning to baseline (Figure 4A). Db RAS mice, however, developed marked albuminuria (at least 6-fold higher than db sham mice) that persisted throughout the observation period (Figure 4A). To determine if basement membrane thickening or podocyte loss (which is some of the characteristic features of diabetic nephropathy) contribute to this transient albuminuria, we performed electron microscopy on the contralateral kidneys of db/db and WT mice at 6 weeks of hypertension. Mean glomerular basement membrane thickness in the contralateral db RAS kidney was increased by 30% after 6 weeks compared to db sham mice (Figure 4B, 4C, 4E), and their glomeruli also showed extensive podocyte foot process effacement (Figure 4D), which was not observed in the contralateral kidney of db sham, WT sham, or WT RAS mice.

### Angiotensin-II induced hypertension does not reproduce the renal injury induced by renovascular hypertension in db mice

**Figure 5:**
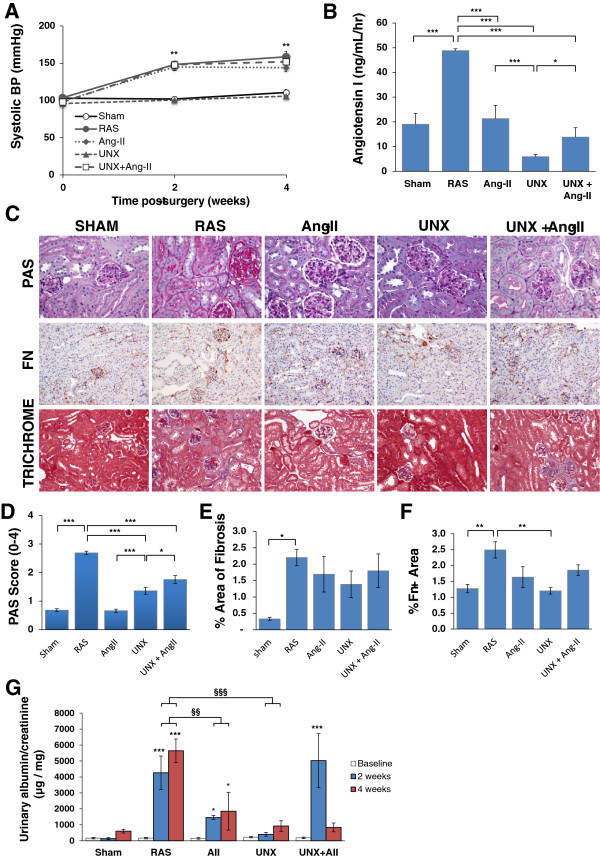
**Angiotensin-II induced hypertension (Ang-II), unilateral nephrectomy (UNX), and combination of both angiotensin-II and unilateral nephrectomy (UNX + Ang-II) unable to re-produce all aspects of injury seen in the contralateral kidney of db RAS mice. A**. Systolic BP were elevated to a similar extent in db RAS, db Ang-II and db UNX + Ang-II (p = ns) and not elevated in the db UNX mice. ** p < 0.01 vs. db sham. **B**. Plasma renin activity (PRA) was elevated only in the db RAS. PRA was assessed by angiotensin-I production. **C**. Representative histological images of contralateral kidney of db RAS and db sham mice, left kidney of db Ang-II mice, and remnant kidney of db-UNX and db UNX+Ang-II mice as stained with periodic acid schiff (PAS), fibronectin, and Masson’s Trichrome. PAS images are taken at 400× while fibronectin and trichrome images are taken at 200×. **D**. Mesangial matrix expansion was seen on db RAS, and to a lesser extent on the db UNX and db UNX + Ang-II, but not on db sham or db Ang-II. **E**. Interstitial fibrosis was increased to a similar extent in all models as assessed by trichrome stained section. **F**. Interstitial fibronectin deposition was increased to a greater extent in db RAS mice compared to all other models. For **B**, **D**, and **F**, * p <0.05 vs. db sham, db Ang-II, db UNX or db UNX + Ang-II. **G**. Urine albumin excretion is highest in db RAS and db UNX + Ang-II mice at 2 weeks post-surgery. * p < 0.05, ** p < 0.01 vs. db sham. § p <0.05 vs. db Ang-II. Albuminuria was measured in spot urine sample and normalized to urine creatinine. Data are presented as means ± SE. Statistical significance was determined by one-way ANOVA followed by Tukey adjusted post-hoc comparison.

A critical role for angiotensin II in the development of chronic renal disease due to etiologies such as diabetes and hypertension has long been recognized [9-13]. We therefore infused db/db mice with angiotensin II (db Ang-II) or PBS for 4 weeks to test the hypothesis that the severe chronic renal damage observed in the contralateral kidney of db RAS mice is primarily due to elevated angiotensin II levels. Db Ang-II mice developed hypertension comparable to that observed in db RAS mice (Figure 5A) despite lower plasma renin content (Figure 5B). Unlike the db RAS mice, the db Ang-II mice showed a minimal increase in mesangial matrix with no evidence of glomerular fibronectin deposition (Figure 5C). The mean glomerular PAS mesangial matrix score in db Ang-II mice was similar to that of db sham mice, whereas that of db RAS mice was over 4-fold higher (Figure 5D). Both db RAS and db Ang-II developed similar degree of tubular atrophy, focal interstitial inflammation and interstitial fibrosis (Figure 5C, 5E), though the db Ang-II mice showed slightly less interstitial fibronectin deposition (Figure 5F).

Despite the lack of mesangial matrix expansion, db Ang-II mice developed significant albuminuria, similar to levels observed in the db RAS mice (Figure 5G). Thus, increased interstitial fibrosis and albuminuria, but not mesangial matrix expansion, can be attributed to angiotensin-II induced hypertension in db/db mice.

### Development of renal injury is accelerated in db RAS than in db/db nephrectomized mice (db-UNX)

Given that angiotensin II infusion in db/db mice failed to produce the lesions observed in db RAS mice, we sought to determine whether increased blood flow to the remaining kidney in mice with unilateral nephrectomy (db UNX) was responsible for the development of mesangial sclerosis, interstitial fibrosis, and tubular atrophy. Unlike db RAS mice, db UNX mice did not develop significant hypertension (Figure 5A), and plasma renin content was lower than that observed in db RAS or db sham (Figure 5B). After 4 weeks, db UNX developed mesangial matrix expansion that was significantly greater than that observed in db sham or db Ang-II mice, but less than in the contralateral db RAS kidney (Figure 5C, 5D). As with db Ang-II, db UNX developed more modest interstitial fibrosis compared to db RAS (Figure 5C, 5E) and showed no increased interstitial fibronectin deposition in comparison to db sham (Figure 5C, 5F). Db UNX developed modest albuminuria, but significantly less than that observed in db RAS mice (Figure 5G).

### The severity of injury in the contralateral db RAS kidney exceeds that induced by a combination of UNx and Angiotensin-II induced hypertension

As angiotensin-II induced hypertension and unilateral nephrectomy replicate only some aspects of injury seen in the contralateral kidney of the db RAS mice, we then sought to determine if the combination would produce the severe injury observed in db RAS mice. We thus infused angiotensin-II into db/db mice subjected to unilateral nephrectomy (db UNX + Ang-II). As with the angiotensin-II infusion alone, db UNX + Ang-II mice developed similar level of hypertension (Figure 5A) with low plasma renin content (Figure 5B). After 4 weeks, we saw a modest increase in the development of mesangial matrix expansion in db uNX + Ang-II mice compared to the db UNX, but lower than the extent of the injury seen in db RAS mice (Figure 5C, 5D). Similarly, we observed an increase in interstitial fibrosis and fibronectin deposition in the db UNX + Ang-II mice compared to the db UNX (Figure 5C, 5E, 5F), but similar to those observed in the AngII group. However, the db UNX + Ang-II mice still developed significantly less fibrosis in comparison to db RAS, indicating other factors that might be contributing to the development of this injury. Interestingly, db UNX + Ang-II mice developed a similar degree of albuminuria as seen in the db RAS mice at 2 weeks (Figure 5F), but returned to baseline levels at 4 weeks.

### Db RAS mice developed greater renal inflammation

**Figure 6:**
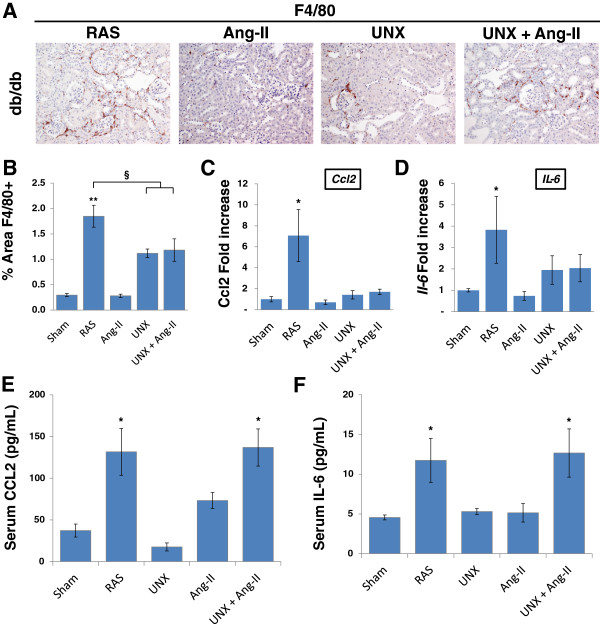
**Db RAS mice experienced higher level of inflammation compared to db mice with Angiotensin-II induced hypertension (Ang-II), unilateral nephrectomy (UNX), or both angiotensin-II and unilateral nephrectomy (UNX + Ang-II). A**. Representative immunostaining of renal macrophages in paraffin section of contralateral or remnant kidneys. Macrophages were stained with anti F4/80 antibody. All images are taken at 200× magnification. **B**. Contralateral kidney of db RAS mice experienced higher increase of renal macrophage infiltration compared to db Ang-II, db UNX, or db UNX + Ang-II. ** p < 0.01 vs. db sham or db Ang-II, § p < 0.05 vs. db UNX or db UNX + Ang-II. Macrophages were stained with F4/80 antibody and quantified with NIS image analysis system. **C-D**. RT-PCR analysis of *Ccl2***(C)** and *Il-6***(D)** mRNA as normalized to 18 s showed that contralateral kidney of db RAS mice produces more inflammatory cytokines and chemokines compared to db Ang-II, db UNX, or db UNX + Ang-II. * p < 0.05 for db RAS vs. all other groups. **E-F**. Serum CCL2 **(E)** and IL-6 **(F)** concentration is elevated to a similar extent in db RAS and db UNX + Ang-II (p = ns). * p < 0.05 vs. db sham, db UNX, or db Ang-II. Data are presented as means ± SE. Statistical significance was determined by one-way ANOVA followed by Tukey adjusted post-hoc comparison.

We and other investigators have shown that the stenotic kidney can become a source of inflammatory cytokines and chemokines [14,17-19] that can cause remote injuries. Therefore, we sought to determine if the db RAS mice experienced higher degree of inflammation in comparison to the control groups. Histological examination showed a significantly higher infiltration of F4/80 renal macrophages in the contralateral kidney of the db RAS mice compared to the other models (Figure 6A, 6B). RT-PCR of *Ccl2* and *Il-6* as marker of inflammation in the contralateral or remaining kidneys of the mice showed significantly higher elevation of both *Ccl2* and *Il-6* mRNA in the db RAS compared to the other models (Figure 6C, 6D). In contrast, both db RAS and db UNX + Ang-II showed similar elevation of serum CCL2 and IL-6.

**Figure 7:**
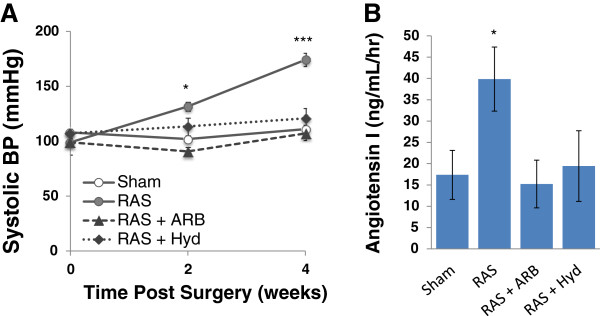
**Both Angiotensin Receptor Blocker (ARB) and hydralazine lower systolic blood pressure in db RAS. A**. Systolic blood pressure measurement of db sham or db RAS mice given no treatment, angiotensin receptor blocker (ARB) or hydralazine (Hyd). **B**. Both ARB and hydralazine treated mice have decreased plasma renin activity at 4 weeks post RAS as assessed by angiotensin I production. * p < 0.05, *** p < 0.001 for db RAS vs. all other groups.

### Reduction of blood pressure ameliorates chronic injury to the contralateral kidney of db RAS mice

**Figure 8:**
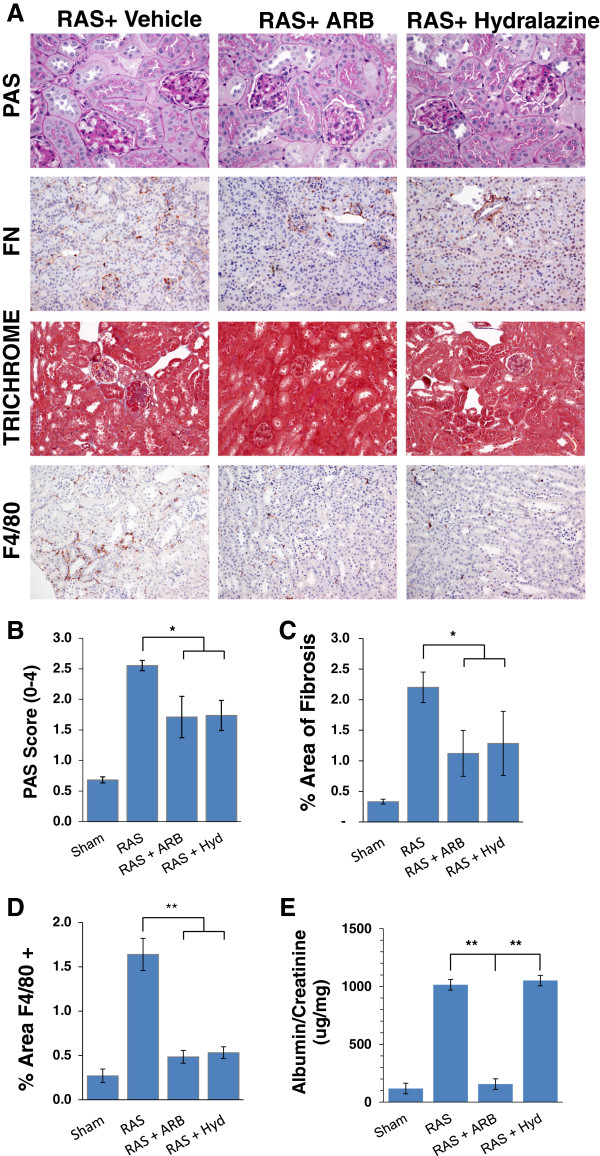
**Both Angiotensin Receptor Blocker (ARB) and hydralazine afford similar attenuation of injury in the contralateral kidney of db RAS. A**. Representative histological images of contralateral kidney of db/db mice undergoing RAS or sham surgery and treated with either ARB or hydralazine (Hyd) as stained with periodic acid schiff (PAS), fibronectin (FN), Masson’s Trichrome, and anti F4/80 antibody. PAS images are taken at 400× magnification while fibronectin, trichrome and F4/80 images are taken at 200× magnification. **B**. Both ARB and hydralazine attenuated mesangial matrix expansion to a similar degree in the contralateral kidney of db mice with RAS. **C**. Both ARB and hydralazine attenuated development of interstitial fibrosis to a similar degree in the contralateral kidney of db mice with RAS. **D**. Both ARB and hydralazine reduced renal macrophage infiltration into the contralateral kidney of db RAS mice. **E**. ARB but not hydralazine attenuated albuminuria in the db RAS mice. * p < 0.05, ** p < 0.01.

To further determine the role of angiotensin II in this process, we sought to determine whether lowering blood pressure by angiotensin-II receptor blocker (ARB) or by hydralazine, which induces vasodilation without direct effects on the renin angiotensin system, would ameliorate renal damage observed in the contralateral kidney of db RAS mice. Treatment of db RAS mice with either ARB or hydralazine was similarly effective in reducing blood pressure to baseline levels (Figure 7A). Both ARB and hydralazine treated mice had no significant elevation of plasma renin content at 4 weeks (Figure 7B). ARB and hydralazine were effective in reducing but not abolishing glomerular mesangial matrix expansion (Figure 8A, 8B), glomerular *de novo* fibronectin expression, interstitial fibrosis (Figure 8C), and reduced influx of macrophages into the contralateral kidney (Figure 8D). However, only ARB reduced urine albumin excretion in db-RAS mice to levels observed in WT-RAS mice (Figure 8E).

## Discussion

A role for hypertension in the development of renal lesions in db/db mice has not been clearly established [20-25]. We found that db sham mice did not develop spontaneous hypertension, while db RAS mice develop hypertension to an extent that is similar to that observed in WT RAS mice, yet associated with transient but more prolonged increases in plasma renin activity and greater renal *Ren1* expression. This persistent increase in plasma renin activity in db RAS mice may reflect interactions between hemodynamic forces associated with renovascular hypertension and the diabetic mileau. Despite similar level of systolic blood pressure, the contralateral kidney of db RAS mice developed chronic renal injury characterized by development of mesangial matrix expansion, interstitial fibrosis, tubular atrophy, and interstitial inflammation, as opposed to the contralateral kidneys in a number of other strains of non-diabetic mice subjected to RAS [5,16,26]. Glomerular histopathologic alterations in the contralateral kidney of db/db mice were striking, and reminiscent of those observed in progressive human diabetic nephropathy, with severe and diffuse mesangial matrix expansion, evident as early as 2 weeks following induction of hypertension. Mesangial matrix expansion consistently was far more extensive than in age-matched db sham mice, and was associated with *de novo* glomerular fibronectin expression. Older db/db mice develop glomerular basement membrane thickening, but quantitative studies in this model have not yet been reported [27-29]. We found an increase of glomerular basement membrane thickness in the contralateral db RAS kidney by six weeks post-surgery, as assessed by morphometric analysis of electron microscopic images, a well-recognized feature of evolving diabetic nephropathy. Glomeruli in these kidneys (but not in WT RAS) showed extensive effacement of visceral epithelial cell foot processes, a morphologic correlate of the progressive albuminuria observed in these mice. At all time-points, urine albumin excretion was significantly greater in db RAS than db sham mice. Based on these observations, we conclude that renovascular hypertension markedly accelerates renal disease progression in db/db mice as characterized by glomerular mesangial matrix expansion, progressive interstitial fibrosis and inflammation, and breakdown of the filtration barrier. This is in accordance with clinical observations indicating that progression of diabetic nephropathy is accelerated in patients with hypertension.

We infused db/db mice with angiotensin II for 4 weeks to address a potential role of angiotensin II induced hypertension on renal architecture in db/db mice. These mice developed hypertension to levels similar to those attained in db RAS mice, yet we observed a minimal increase in mesangial matrix deposition and no evidence of d*e novo* glomerular fibronectin deposition. Nevertheless, db Ang-II developed albuminuria similar to that observed in db RAS mice and to that reported following angiotensin II infusion to non-diabetic mice [30,31]. Taken together, these observations suggest that the progressive and bilateral renal injury in db RAS mice is not mechanistically related to elevated angiotensin II levels alone, although angiotensin II plays a major role in development of albuminuria in this model [7,8]. This finding underscores a critical role for activation of the renin-angiotensin system in the development of albuminuria and provides a therapeutic rationale for the widespread use of renin-angiotensin inhibitors in treatment of chronic kidney disease.

We then sought to determine whether hyperfiltration associated with unilateral nephrectomy may underlie the progressive renal damage observed in the contralateral db RAS kidney. Unlike db RAS or db Ang-II mice, db UNX did not develop significant hypertension. Db UNX also did not develop increased urine albumin excretion that was observed in the db RAS or db Ang-II. However, as shown before, db/db mice with unilateral nephrectomy did develop greater glomerular mesangial matrix expansion than age-matched db/db mice with two kidneys [8,32], although its extent was less than that of db RAS mice. Although few investigators have directly reported the extent of interstitial fibrosis in this model, db/db mice evaluated at 14 – 18 weeks post UNX exhibited a modest increase in interstitial inflammation, interstitial volume, and number of tubules showing dilation or atrophy [8,32,33]. In the current study, we find that db UNX mice, in striking contrast to db RAS mice, do not develop significant interstitial fibrosis or tubular atrophy at 4 weeks post UNX. Therefore, glomerular mesangial matrix expansion in db/db mice can be attributed at least in part to hemodynamic factors associated with hyperfiltration, whereas elevation of blood pressure appears to play a major role in development of albuminuria in db/db mice.

As Angiotensin-II induced hypertension and UNX alone only recapitulate some features of renal injury seen in the contralateral kidney of db RAS mice, we combined both in db/db mice. Remaining kidneys of db UNX + Ang-II mice developed all the features seen in the db RAS mice, namely mesangial expansion, interstitial fibrosis, tubular atrophy, and albuminuria, but the severity of injury observed in the contralateral kidney of db RAS mice was greater than that of db UNX + Ang-II mice.

To examine if hypertension was necessary for the development of progressive renal fibrosis in the contralateral kidneys of db/db mice, we treated them with ARB or the vasodilator hydralazine, which lowered blood pressure to levels similar to those observed in db sham mice without significant changes in plasma renin activity. Reduction of blood pressure was effective in reducing mesangial matrix expansion, fibronectin expression, interstitial fibrosis, and tubular atrophy in the contralateral kidney of db RAS mice. However, urine albumin excretion was significantly reduced by ARB only. Therefore, we conclude that hypertension plays an essential role for the development of chronic renal lesions in the contralateral kidney of db/db mice subjected to RAS, while increase level of angiotensin II plays a role in the development of albuminuria. Interestingly, while both drug treatments attenuate the development of renal injury, both do not abolish it. Given the less severe injury observed in the db UNX + Ang-II, these results point to some other factor independent of blood pressure elevation and hyperfiltration process that is mediated by the stenotic kidney, possibly by the activated RAAS.

We and other investigators have shown that the stenotic kidney experienced considerable oxidative stress [14,17] and produced substantial level of inflammatory cytokines [18,19]. Indeed, in comparison to the other models, contralateral kidney of db RAS exhibited significantly higher expression of the inflammatory chemokine CCL2 and the inflammatory cytokine IL-6, both of which represent prognostic of development of renal injury [34-36]. Nonetheless, db RAS showed similar increased in serum CCL2 and IL-6 to db UNX + Ang-II. However, although serum levels of CCL2 might be elevated in diabetic patients, they are not associated to the development of albuminuria, renal macrophage influx, or interstitial fibrosis [37-39]. Instead, both urine CCL2 and IL-6 excretion – reflecting production of these inflammatory molecules within the kidney itself – have been shown to correlate significantly with progression of renal injury [37,38,40]. Furthermore, increased albuminuria may itself aggravate tubular injury and accelerate development of renal injury by increasing tubular CCL2 and IL-6 production [41,42].

## Conclusion

In summary, renovascular hypertension accelerates development of diabetic renal injury in db/db mice that recapitulates many of the features of chronic renal disease in subjects with diabetes and hypertension and markedly accelerated the progression of chronic renal disease. As hypertension induced by angiotensin II infusion was not sufficient to reproduce these lesions, we believe that interactions between the diabetic milieu and hemodynamic forces associated with hyperfiltration were necessary to produce progressive renal disease in db/db mice. Although combination of Angiotensin-II infusion and unilateral nephrectomy are able to replicate many features of injury observed in the db RAS, the db RAS model is likely more physiologically relevant to the development of diabetic nephropathy in patients with both diabetes and RAS, and will allow the development of mechanistic studies to identify critical pathways related to inflammation, fibrosis, oxidative stress, and cell cycle regulation that are responsible for the development and progression of diabetic renal disease.

## Competing interests

The authors declare that they have no competing interests.

## Authors’ contributions

SPH and JPG conceived the project. SPH, BEK and JPG designed the methods and the experiments. BEK performed all the animal surgeries. SPH carried out the laboratory experiments, analyzed the data, and interpreted the results. SPH and JPG wrote the manuscript. LOL and SCT gave technical support, conceptual advice, and critical review of the manuscript. All authors read and approved the final manuscript.

## Pre-publication history

The pre-publication history for this paper can be accessed here:

http://www.biomedcentral.com/1471-2369/15/58/prepub
